# A twin-tower model using MRI and gene for prediction on brain tumor patients’ response to therapy

**DOI:** 10.1093/bioadv/vbaf041

**Published:** 2025-03-04

**Authors:** Qiyuan Lyu, Fumie Costen

**Affiliations:** Electrical and Electronic Engineering Department, The University of Manchester, Manchester M13 9PL, United Kingdom; Electrical and Electronic Engineering Department, The University of Manchester, Manchester M13 9PL, United Kingdom

## Abstract

**Motivation:**

Glioma is the most prevalent and aggressive primary brain tumor, with a poor prognosis of patients and a high mortality rate. Standard treatment of surgery, radiation, and chemotherapy may not be effective for some patients as they suffer from a stable progression of disease after treatment. Hence, it is crucial to predict the patient’s response to therapy as a guide for the treatment plan. In this paper, we propose a multimodal model based on both magnetic resonance imaging and genomic data. As the dataset has a majority of single-modality samples with a few ratios of multi-modality samples, we propose a twin-tower architecture to solve the unimodal dominance issue and fully use the single-modality data.

**Results:**

The proposed architecture comprises an image encoder and a gene encoder trained on the single-modality samples for feature extraction, along with a classification head trained on multi-modality samples. In this way, all the single-modality samples can be beneficial to the whole model, and the need for the multi-modality is diminished. The proposed model outperforms the comparison methods across all metrics, achieving an accuracy of 85% on the cross-validation. The ablation experiment comparing the proposed architecture with single-modality models reflects the effectiveness of the proposed twin-tower architecture.

**Availability and implementation:**

The proposed model exhibits excellent scalability and can accommodate the integration of additional modalities without the requirement of too many multi-modality samples.

## 1 Introduction

Gliomas, a heterogeneous group of neuroepithelial tumors arising from the glial cells, are the most common and deadly primary brain tumors (Miller *et al.* 2021). The prognosis of glioma is poor, with a median survival of 14.6 months for glioblastoma (GBM), the most aggressive form of glioma, and 7.3 years for lower-grade glioma (LGG) (Louis *et al.* 2021). Currently, the standard treatment for glioma includes surgical resection, radiation, and chemotherapy (Qi *et al.* 2023). However, some patients may suffer from post-treatment tumor progression or recurrence. Generally, tumor progressive diseases signify treatment failure, while post-treatment effects, radiation necrosis, and pseudoprogression indicate a positive response to treatment (Parvez *et al.* 2014). Accurately predicting the patient’s response to therapy is critical for guiding treatment plans and ultimately improving the patient’s prognosis.

Most of the current methods using medical imaging to predict the patient’s response to treatment are based on the handcrafted radiomic features extracted from medical imaging, such as the shape, texture, and intensity of the region of brain tumor and developed into machine learning models, such as Support Vector Machine (SVM) and Random Forest (RF) (Siakallis *et al.* 2021, Zhou *et al.* 2022, de Godoy *et al.* 2023). In addition to medical imaging, genomic characteristics offer a novel perspective for the diagnosis, treatment management, and early prediction of glioma. The current methods using genomic data predominantly rely on statistical analysis and machine learning methods to identify specific gene expressions associated with brain tumors and potential targeted treatment (Menyhárt *et al.* 2021, Arrieta *et al.* 2023, Jang *et al.* 2023).

MRI images of brains can intuitively reflect the features of brain tumors, such as the location, size, shape, etc., which is highly relevant to the patient’s response to treatment. Genetic data provide insights into tumor biology, including pathways involved in proliferation, invasion, and treatment resistance, while MRI captures spatial and morphological characteristics of the tumor *in vivo*. The integration of gene expression and MRI data in multi-modal analyses offers a powerful approach to unraveling the biological underpinnings of GBM. By combining these data modalities, understanding of tumor heterogeneity, identify prognostic biomarkers is enhanced. Therefore, it is necessary to delve into genomic data together with MRI data to more comprehensively assess the patient’s response to treatment. Patel *et al.* (2021) proposed a model that combined clinical characteristics, genomic data and quantitative imaging features to predict the patient’s response to therapy.

The current multimodal models face several challenges. On the one hand, the existing multimodal models generally employ machine learning methods to handle genomic data and perform the radiomic and genomic features fusion, while deep learning approaches demonstrate advantages in terms of performance. On the other hand, current multimodal methods generally merge the handcrafted features extracted from image and genomic data, which requires samples to possess information from multi-modalities to fulfill data alignment, however, for samples with single modality, existing models lack the capability of features fusion. Hence, there is a large amount of single-modality samples, which cannot be fully utilized in the current models. Since obtaining sufficient multimodal samples is challenging, effectively leveraging the vast amount of single-modality samples becomes a critical factor for improving the accuracy of this task. Hence, a multimodel deep-learning-based architecture based on MRI and genomic data is worth exploring.

In this paper, we propose a multimodal architecture that utilizes both MRI and genomic data for predicting patients’ responses to therapy. The purpose of the proposed model is to make full use of single-modality data and avoid the unimodal dominance issue as the dataset has a majority of single-modality samples. The proposed model is designed as a twin-tower architecture, comprising an image encoder and a gene encoder for feature extraction, and a classification head for feature fusion and classification. The image and gene encoder can be trained independently on the single-modality samples and then transferred to the multi-modality samples to train the classification head. The proposed twin-tower architecture outperforms the baseline methods across all metrics, such as accuracy, precision, recall, and F1-score. We conduct an ablation experiment to evaluate the effectiveness of the proposed twin-tower architecture compared to the single-modality models. Moreover, the proposed model exhibits excellent scalability and can accommodate the integration of additional modalities without the requirement of too many multi-modality samples.

## 2 Method

### 2.1 Dataset

The dataset used in our study is derived under two publicly available datasets from The Cancer Genome Atlas (TCGA) dataset, an open-source, open-access information resource, namely, The Cancer Genome Atlas Glioblastoma Multiforme (TCGA-GBM) (Scarpace *et al.* 2016) and The Cancer Genome Atlas Lower Grade Glioma (TCGA-LGG) (Pedano *et al.* 2016), which provide a diverse range of data types, including MRI, genomic data, and clinical information.

In particular, the MRI data is sourced from the pre-operative_TCGA-GBM (Bakas *et al.* 2017a) and pre-operative_TCGA-LGG (Bakas *et al.* 2017b) dataset, which provides pre-processed MRI data obtained and then pre-processed from the relative TCGA datasets. These two datasets consist of various modalities of MRI data, including T1-weighted (T1), T2-weighted (T2), T1-weighted after contras (T1ce), and (Fuid-Attenuated Inversion Recover) FLAIR, as well as segmentation data of brain tumors. Standardized pre-processing of the MRI data was performed by the two datasets, starting with a co-orientation to the left-posterior-superior coordinate system, co-registering to the same anatomical template, followed by resampling to a uniform 1 mm^3^ voxel resolution and skull-stripping to remove the skull in MR images, making the tumor region more conspicuous and mitigating potential facial reconstruction/recognition of the patients (Bakas *et al.* 2017c). We utilized the maximum voxel layer sampling method to extract 2D slices from 3D MRI images along the transverse axis. Each slice of the input MR images (T1, T2, T1ce, and FLAIR) and the segmented data has a resolution of 240×240.

Regarding the genomic data, we obtained the RNA sequence (RNA-seq) data from TCGA-GBM and TCGA-LGG datasets in the form of “Gene Expression Quantification,” based on which an initial gene expression matrix was generated from the calculation of the expression levels for each gene. We employed the gene annotation file “Homo_sapiens.GRCh38.105.chr.gtf.gz” to convert features in the gene expression matrix (Ensemble Transcript ID) to stable gene IDs and remove the repeated gene IDs. In total, 19 196 gene IDs were obtained in each sample.

The genomic and clinical data from TCGA-GBM, TCGA-LGG datasets and the MRI data from the relative preoperative MRI datasets were combined into one dataset. We transform the patients’ response task into a binary classification problem, where the labels represent “1” for positive response to therapy and “0” for negative response to therapy. Overall, we have 95 samples with MRI data only, 452 samples with genomic data only, and the remaining 50 samples with all modalities. Samples with a single modality were used to train the models specific to that particular modality with the ratio of training and testing set as 80:20. The 50 samples with all modalities were partitioned into a training set for transferring learning and a testing set with a ratio of 3:2.

### 2.2 Architecture

**Figure 1.**
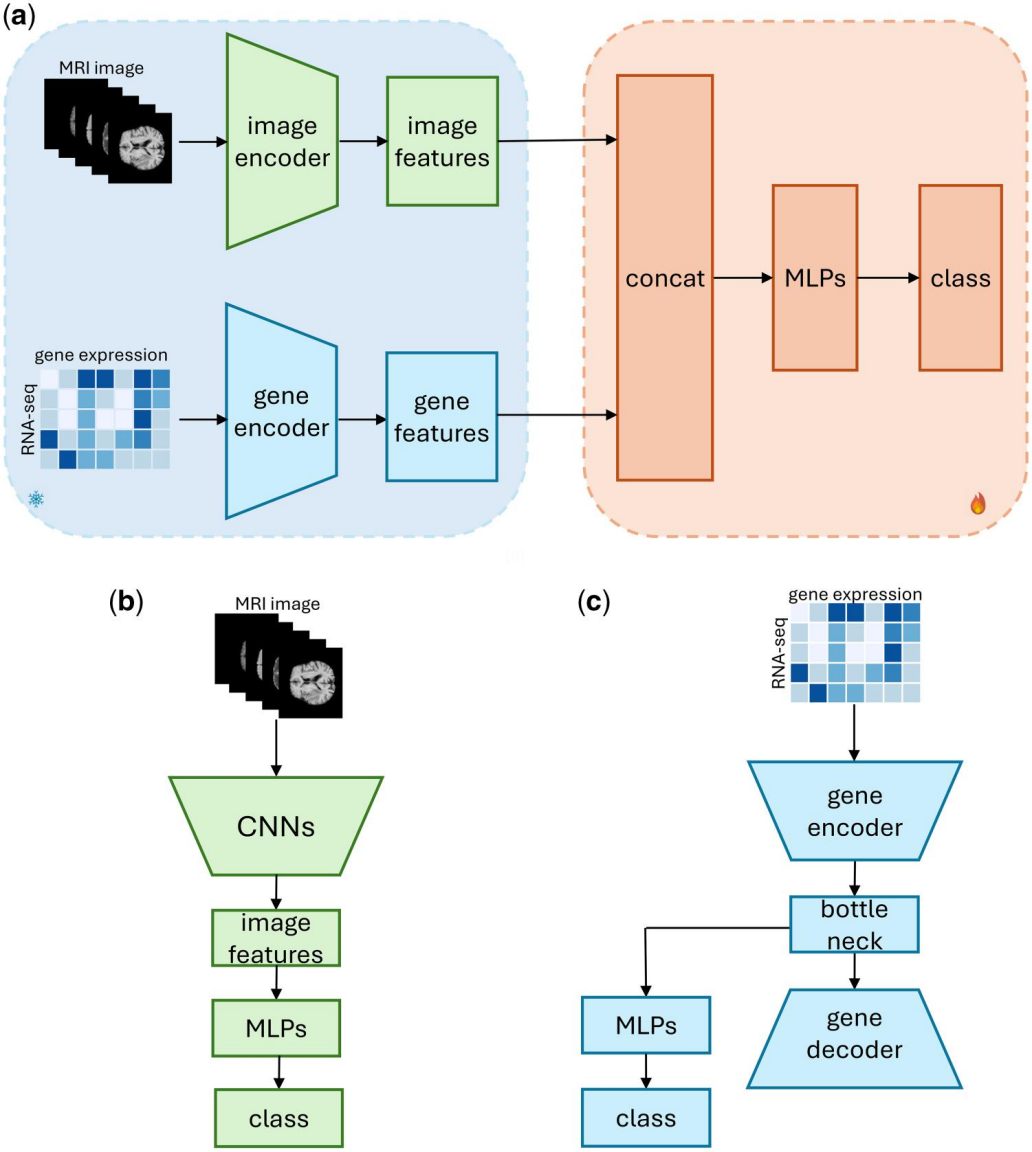
Overflow of the twin-tower framework. (a) The main architecture based on a twin-tower framework comprises an image encoder and a gene encoder, which are trained from single-modality samples, for feature extraction, and MLPs for classification. (b) The image encoder uses CNN and MLP to extract image features from MRI. The image encoder is a low-complexity CNN-based architecture from our previous paper (Lyu *et al.* 2024) (c) The gene encoder is an AutoEncoder architecture. The encoder of AE model consists of two dense layers to compress the gene data, while the decoder reverses the encoder to reconstruct the gene data from the compressed features inside the bottleneck. The image encoder and gene encoder will be frozen when transferring the model on the multimodel samples while merely the MLPs will be tuned.

Based on the analysis in the Dataset section, it can be concluded that multimodal samples are in the minority, while the majority of samples merely possess data from a single modality (MRI or gene). With the purpose of addressing the issue of unimodal dominance in multimodal datasets, we develop a twin-tower structure that extracts the single-modality features parallelly from two single-modality models and a concatenate layer for the image/gene feature fusion. The schematic diagram of our proposed twin-tower structure is shown in Fig. 1a. We build up the image tower and gene tower independently based on two encoders to extract the feature from image and gene samples and train the two towers based on the samples that consist of a single modality. Following that, the features (image features and gene features) extracted from the two single-modality towers are merged into MLP layers, for modality fusion and classification purposes.

We utilize a low-complexity CNN-based architecture (Lyu *et al.* 2024) for the image encoder, as shown in Fig. 1b, where three convolutional blocks consisting of convolution, pooling, and activation layers (ReLU) and two dense layers with 32 and 16 nodes are employed for the feature extraction from MRI. The input MRI data includes T1, T2, T1ce, FLAIR, as well as the segmented data of the tumor region. For gene data, we employ an AutoEncoder model to compress and extract features from raw RNA sequence, which is shown in Fig. 1c. The encoder of our AE model consists of two dense layers with 64 and 32 nodes, respectively, to compress the gene data, while the decoder reverses the encoder, with 32 and 64 nodes sequentially, to reconstruct the gene data from the compressed features inside the bottleneck. We utilize the mean squared error as the reconstruction loss for optimization of the AE model. Two dense layers with 64 and 16 nodes are connected to the bottleneck for the classification task after the AE is trained. The two towers used for feature extraction with 32 features obtained from the gene encoder and 16 features obtained from the image encoder, are then scaled separately with Gaussian normalization with mean of 0 and variance of 1, and connected with three dense layers with 64, 128, and 16 nodes, respectively, for classification, followed by a dense layer for output. When conducting transfer learning on the multi-modality samples, the single-modality encoders are frozen and only the concatenate and classification head are trained on the multi-modality samples.

Through this architecture, we maximize the utilization of single-modality samples to reach a high capability of feature extraction and a better modal fusion and transfer effects on the whole dataset without the need for a large number of multi-modality samples. This architecture significantly enhances the scalability of the model.

## 3 Results

### 3.1 Experimental setup

To solve the issue of unimodal dominance, the proposed twin-tower model underwent a two-stage training procedure. In the first stage, we started with training the two single-modality towers on the single-modality samples, therefore, an image tower and a gene tower were obtained as image and gene encoder, respectively. After that, the output features from the two single-modality towers were merged into the feature fusion layer after a separate normalization. The second stage would transfer the scaled features on the multi-modality samples to train the feature fusion layer and the classification head, while the two encoders were frozen. The whole two-stage training had hyperparameters as shown in Table 1. The hyperparameters for the first stage were obtained from the grid search method based on the validation set, while the hyperparameters for the second stage were set based on the first stage. We used stochastic gradient descent (SGD) as the optimizer for the image encoder and transfer learning, and Adam for the gene encoder, with a learning rate of $2\times 10^{-5}$ for the image encoder, $1\times 10^{-4}$ for the gene encoder and the transfer learning, and a decay rate of $2\times 10^{-7}$ for all the models. For the loss functions, we used cross entropy for the image encoder and transfer learning and the mean squared error (MSE) for the gene autoencoder. The epoch was set to 20 for the image and gene encoders and 10 for the transfer learning as it was only transferred on a minority of multi-modality samples.

**Table 1.** Hyperparameters for the proposed model.

Hyperparameters	Value		
	Image encoder	Gene encoder	Transfer
Optimizer	SGD	Adam	SGD
Momentum	0.9	0.9	0.9
Loss function	cross entropy	MSE	cross entropy
Learning rate	$2\times 10^{-5}$	$1\times 10^{-4}$	$1\times 10^{-4}$
Decay rate	$2\times 10^{-7}$	$2\times 10^{-7}$	$2\times 10^{-7}$
Epoch	20	20	10
Batch size	16	16	16

### 3.2 Results and analysis

We chose several references (Sun *et al.* 2021, Jalalifar *et al.* 2022, de Godoy *et al.* 2023, Sadique *et al.* 2024) as baseline models for comparison. (Jalalifar *et al.* 2022) proposed a fusion model that used the InceptionResentV2 network to extract distinct features from each MRI slice and transformer network incorporate spatial dependencies between MRI slices based on the T1 and FLAIR image and clinical information. (Sadique *et al.* 2024) includes multiresolution radiomic feature (MRF) extraction extracted from mpMRI (T1, T2, T1ce, FLAIR) and selected with statistical significance testing, followed by a CatBoost classifier, a method of gradient boosting decision trees class. (de Godoy *et al.* 2023) proposed a multiparametric MRI model consisting of predictive probabilities of tumor progression computed from diffusion and perfusion MRI-derived parameters. (Sun *et al.* 2021) investigated a machine learning model combining clinical characteristics, and texture features extracted from T1ce.

We measured accuracy, precision, recall, and F1-score to evaluate the proposed model, which are all calculated on the three-fold cross-validation. Accuracy, precision, recall and F1-score denote the proportion of proportion of correct predictions among the total number of predictions, the proportion of true positive predictions among all positive predictions, the proportion of true positive predictions among all actual positive cases and the harmonic average of precision and recall, respectively. The calculation of accuracy, precision, and recall are shown as (1–4).

(1) $$\text{Accuracy}\;=\;\frac{\text{TP}+\text{TN}}{\text{TP}+\text{FP}+\text{FN}+\text{TN}}$$

 

(2) $$\text{precision}\;=\;\frac{\text{TP}}{\text{TP}+\text{FP}}$$

 

(3) $$\text{recall}\;=\;\frac{\text{TP}}{\text{TP}+\text{FN}}$$

 

(4) $$F1=2\cdot \frac{\text{precision}•\text{recall}}{\text{precision}+\text{recall}}$$

where True Positive (TP) is the number of positive examples correctly predicted as positive, True Negative (TN) is the number of negative examples correctly predicted as negative, False Positive (FP) is the number of negative examples wrongly predicted as positive, and False Negative (FN) is the number of positive examples wrongly predicted as negative.

As shown in Table 2, our proposed model demonstrated a significant performance improvement over several baseline models. Specifically, our proposed twin-tower model achieved the highest accuracy of 85%, which is 2.5% higher compared to the InceptionResNet model Additionally, in terms of precision, recall and F1-score, our proposed model reached 93.3%, 87.5%, and 90.3%, respectively, indicating an enhanced classification capability and a lower rate of false positives. These experimental results verify the effectiveness of our proposed model in this task and further underscore our proposed model’s potential value in medical applications. Theoretically, image features such as location, size, and shape, are contributive to the diagnosis of brain tumors and the prognosis of patients. Hence, an image-based model can have a good performance in the task of prediction of patients’ response to treatment. However, pathologically, once a tumor starts to spread and metastasize, which frequently occurs for glioma, the recurrence or stable progression may happen after treatment. This kind of information is not able to be obtained from MRI, while the gene expression level can reflect it. Hence, a multimodal architecture combining the MRI and genomics data can outperform the single-modality methods.

**Table 2.** Comparison between our proposed model and the baseline.

Model/Metrics	Accuracy	Precision	Recall	F1-score
**Proposed model**	**0.85**	**0.933**	**0.875**	**0.903**
InceptionResNet Jalalifar *et al.* (2022)	0.825	87	0.765	0.814
MRF Sadique *et al.* (2024)	0.801	0.8	0.85	0.824
mpMRI model de Godoy *et al.* (2023)	0.765	0.889	0.727	0.80
RF Sun *et al.* (2021)	0.728	0.613	0.784	0.688

The top one performance of each columns is in bold.

### 3.3 Ablation experiment

We conducted an ablation experiment to evaluate the effectiveness of the twin-tower architecture versus the two single-modality models and the concatenate layer versus the mixture-of-expert (MoE) layer (Jiang *et al.* 2024). In this comparison, we used the single-modality samples for training the single-modality towers and the abovementioned transfer learning method for training the twin-tower model, and the same testing set to evaluate all the models.

**Figure 2.**
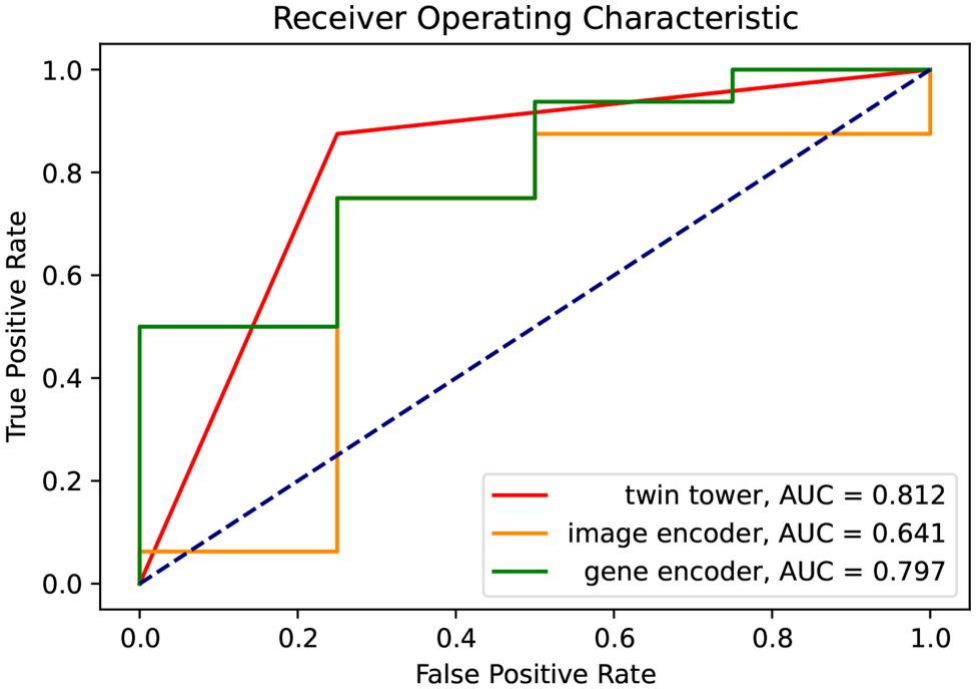
ROC curve of the twin-tower model compared to the image encoder and gene encoder.

We compared the twin-tower model with the two single-modality models, as shown in Table 3 and the ROC curve in Fig. 2. The twin-tower architecture outperformed the two single-modality models, with an accuracy of 85%, while the image-only model and gene-only model achieved an accuracy of 74% and 72.3%, respectively. From precision it can be concluded that the image and gene models classify positive and negative samples more accurately, respectively. After employing the twin-tower structure, the model effectively utilizes the strengths of two single-modality models, resulting in a significant improvement in precision. The twin-tower architecture also performed better in recall, and F1-score than the two single-modality models, indicating that this architecture is more capable of predicting the patient’s response to therapy than the two single-modality models. In the comparison of the two fusion methods, the concatenate layer outperformed the MoE layer, with an accuracy of 85%, while the MoE layer achieved an accuracy of 75%. However, this is not a fair comparison, as the MoE layer requires a large number of multimodal samples to train the model. When more modalities are introduced into the model, the MoE layer may be more effective as the number of experts in the MoE layer can be increased to handle a large number of modalities.

**Table 3.** Results of the Ablation experiment.

Model/Metrics	Accuracy	Precision	Recall	F1-score
**Twin-tower model**	**0.85**	**0.933**	**0.875**	**0.903**
Image model only	0.74	0.762	0.865	0.81
Gene model only	0.723	0.630	0.725	0.674
**Concatenate**	**0.85**	**0.933**	**0.875**	**0.903**
MoE	0.75	0.9	0.69	0.78

The top one performance of each columns is in bold.

**Figure 3.**
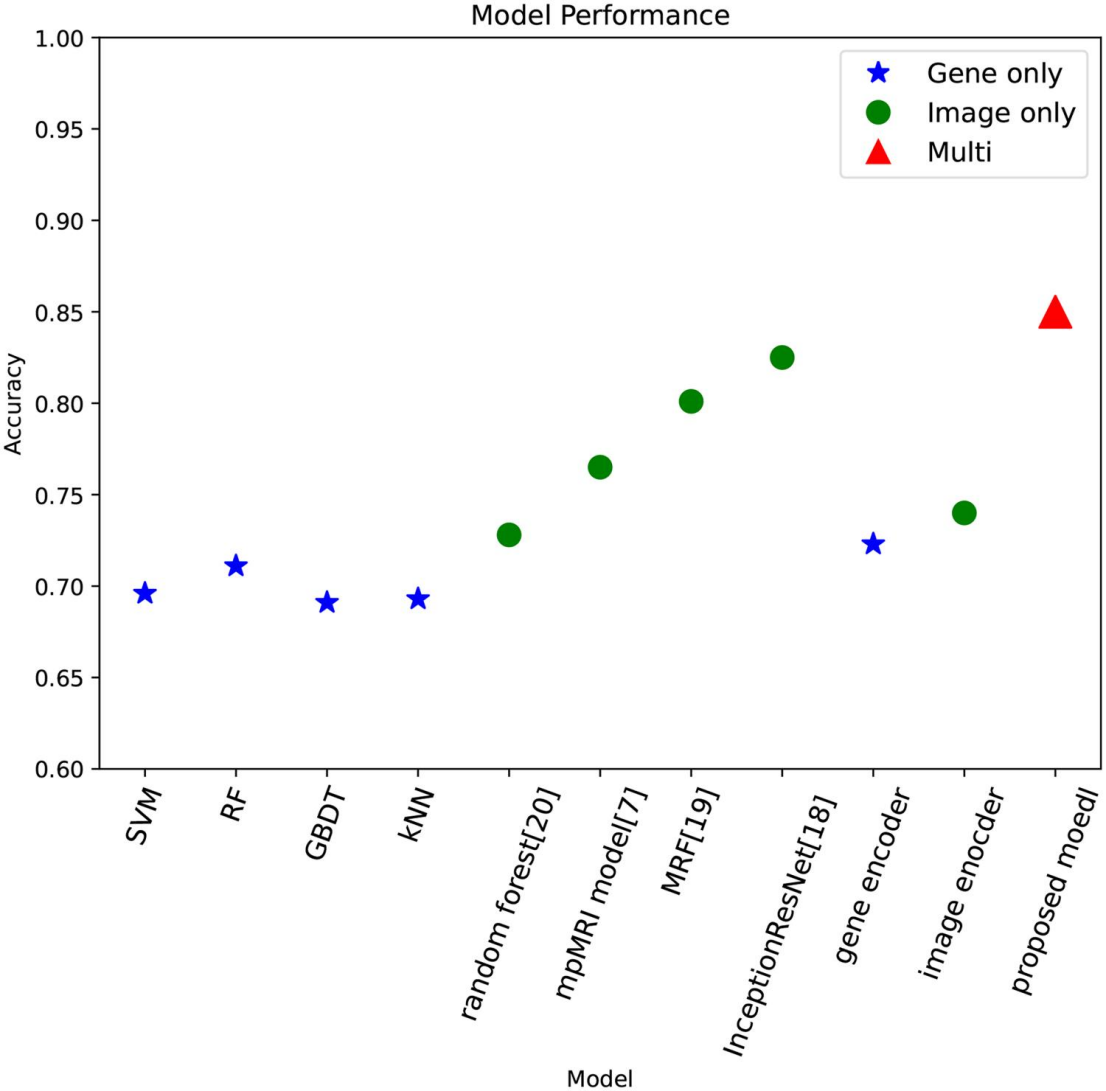
Performance of the proposed model compared to the baseline methods. The stars, cirlces, and triangles represent the model with gene only, image only and multi-modality, respectively.

In the model selection for genomic data, we compared the performance of the AE model against the traditional machine learning methods, including SVM, RF, Gradient Boosting Decision Tree (GBDT), and k-nearest Neighbors (kNN), and PCA for feature compression before the classifications with machine learning methods. The results of AE the machine learning methods are as shown in Table 4. Compared to the machine learning methods, AE achieved an accuracy of 72.3%, outperforming the SVM, RF, GBDT, and kNN, which achieved an accuracy of 67.6%, 67.1%, 65.6%, and 64.3%, respectively. PCA improved the performance of the traditional machine learning methods, with an accuracy of 70.6%, 69.8%, 68.6%, and 69.6% for SVM, RF, GBDT, and kNN, respectively, but still falls short compared to AE. The comparison of twin-tower architecture against baselines in accuracy is shown in Fig. 3.

**Table 4.** Results of AE against machine learning method with and without PCA.

Models		Accuracy	Precision	Recall	F1-score
**AE**		**0.723**	0.630	**0.725**	0.674
SVM		0.676	0.681	0.676	0.651
RF		0.671	0.672	0.671	0.652
GBDT		0.656	0.650	0.656	0.643
kNN		0.643	0.633	0.643	0.622
**PCA+**	SVM	0.706	**0.713**	0.706	**0.690**
	RF	0.698	0.704	0.698	0.684
	GBDT	0.686	0.684	0.686	0.676
	kNN	0.696	0.696	0.697	0.685

The top one performance of each columns is in bold.

## 4 Discussion

In this paper, we propose a twin-tower architecture for predicting the patient’s response to therapy based on MRI and genomic data. The proportion of multimodal samples in the dataset is quite small, with the majority of samples consisting of either image or gene modality. To address this unimodal dominance issue, the proposed twin-tower architecture features two single-modality towers for extracting features from MRI and genomic data, respectively. These features are then combined into a feature fusion layer.

We trained the image tower and gene tower with the MRI-only samples from the pre-operative_TCGA-GBM and pre-operative_TCGA-LGG datasets and gene-only samples from the TCGA-GBM and TCGA-LGG datasets, respectively. The feature fusion layer along with the classification head is trained on the multimodal samples using transfer learning, while the two single-modality towers are frozen. The single-modality tower for MRI was built based on a low-complexity CNN-based architecture, while that for gene data was built based on an AE model.

The performance of the proposed twin-tower model outperforms the baseline methods with an accuracy of 85% through cross-validation. The proposed model also had a better performance than the baseline models in terms of precision, recall, and F1-score. This improved performance indicates that the proposed twin-tower architecture is more capable of predicting the patient’s response to therapy than the comparisons. In the ablation experiments, we compared the proposed twin-tower architecture with the two single-modality models and the concatenate layer with the MoE layer. Based on the results of precision, it can be concluded that the image and gene models classify positive and negative samples more accurately, respectively. After employing the twin-tower structure, the model effectively utilizes the strengths of two single-modality models, leading to a significant improvement in precision.

When additional modalities, such as computerized tomography (CT) and biomarkers, are introduced into the model, it is sufficient to train the single-modality model separately for feature extraction on the new modality and the network for modalities fusion, without retraining the feature extraction layers of existing modalities. With more types of data integrated into the proposed architecture, the MoE layer may have an improvement as it is suitable for dealing with various types of data.

## 5 Conclusion

In this paper, we propose a twin-tower architecture to predict the patient’s response to therapy, with MRI and gene data. To utilize the single-modality samples to the maximum extent, the proposed two-tower architecture consists of two single-modality models and a feature fusion layer. The two single-modality models are trained from the single-modality samples for feature extraction, which are merged into the feature fusion layer for classification. The feature fusion layer and the classification head are then trained on the multimodal samples by transfer learning, while the two single-modality models are frozen. The single-modality model for MRI was built based on a low-complexity CNN-based architecture, while that for gene data was built based on an AE model. The key advance with this designed architecture is that we overcame the issue of unimodal dominance in multimodal datasets and achieved a high performance without using too many multimodal samples. In the meantime, this architecture has the capability of scalability for more modalities introduced into the proposed model.

We collected MRI data from the pre-operative_TCGA-GBM and pre-operative_TCGA-LGG datasets and gene data from the TCGA-GBM and TCGA-LGG datasets and combined them into one dataset. The single-modality samples in our dataset consist of 95 samples with MRI data, 452 samples with gene data, and 50 samples with all modalities. We trained the image encoder and gene encoder with the MRI-only and gene-only samples, respectively, and then transferred the encoders to the multi-modality samples to train the feature fusion layer and the classification head. We compared the performance of the proposed twin-tower model with baseline methods and the proposed method outperformed them with an accuracy of 85% through cross-validation. The proposed model also has a better performance than the comparisons in precision, recall, and F1-score. The ablation experiment reflected the effectiveness of the designed twin-tower architecture compared to the single-modality models. This model can help in formulating personalized treatment plans, providing more accurate prognostic assessments, and identification of suitable treatments for patients.

### 5.1 Key points

- A twin-tower architecture using MRI and genomic data is proposed, which can make full use of single-modality data and mitigate the unimodal dominance issue.
- Compared to the baseline models using MRI only and gene only, the proposed model has a better performance in all metrics.
- The usage of twin-tower architecture improves the scalability of the model and can accommodate the integration of additional modalities.

### 5.2 Future work

This paper combines the two datasets and uses the pre-processing MRI data, which consists of 502 samples in total. The scale of the number of data is insufficient to demonstrate the absolute effectiveness of the model in clinical applications. Hence, it is necessary to collect more data to further evaluate the proposed model. Apart from the number of data, we would like to expand the modality of samples, such as CT or biomarkers, to further benefit the performance of the proposed model.

## Data Availability

This paper utilizes the clinical data from TCGA-GBM, TCGA-LGG and pre-processed MRI data from pre-operative_TCGA-GBM and pre-operative_TCGA-LGG. The datasets can be downloaded online from: TCGA-GBM: Scarpace, L., Mikkelsen, T., Cha, S., Rao, S., Tekchandani, S., Gutman, D., Saltz, J. H., Erickson, B. J., Pedano, N., Flanders, A. E., Barnholtz-Sloan, J., Ostrom, Q., Barboriak, D., & Pierce, L. J. (2016). The Cancer Genome Atlas Glioblastoma Multiforme Collection (TCGA-GBM) (Version 4) [Data set]. The Cancer Imaging Archive. https://doi.org/10.7937/K9/TCIA.2016.RNYFUYE9 TCGA-LGG: Pedano, N., Flanders, A. E., Scarpace, L., Mikkelsen, T., Eschbacher, J. M., Hermes, B., Sisneros, V., Barnholtz-Sloan, J., Ostrom, Q. (2016). The Cancer Genome Atlas Low Grade Glioma Collection (TCGA-LGG) (Version 3) [Data set]. The Cancer Imaging Archive. https://doi.org/10.7937/K9/TCIA.2016.L4LTD3TK pre-operative_TCGA-GBM: Bakas S, Akbari H, Sotiras A, Bilello M, Rozycki M, Kirby J, Freymann J, Farahani K, Davatzikos C. (2017). Segmentation Labels for the Pre-operative Scans of the TCGA-GBM collection [Data set]. The Cancer Imaging Archive. DOI: 10.7937/K9/TCIA.2017.KLXWJJ1Q Bakas S, Akbari H, Sotiras A, Bilello M, Rozycki M, Kirby J, Freymann J, Farahani K, Davatzikos C. (2017) Segmentation Labels and Radiomic Features for the Pre-operative Scans of the TCGA-LGG collection [Data Set]. The Cancer Imaging Archive. DOI: 10.7937/K9/TCIA.2017.GJQ7R0EF
